# Response to Mei et al. regarding the incidence and predictors of hospitalization for heart failure among patients with stable atherosclerosis in the TRA 2°P‐TIMI 50 trial

**DOI:** 10.1002/clc.23972

**Published:** 2023-01-24

**Authors:** Benjamin L. Freedman, David D. Berg, Benjamin M. Scirica, Erin A. Bohula, Erica L. Goodrich, Marc S. Sabatine, David A. Morrow, Marc P. Bonaca

**Affiliations:** ^1^ Department of Medicine, Harvard Medical School Beth Israel Deaconess Medical Center Boston Massachusetts USA; ^2^ TIMI Study Group, Brigham and Women's Hospital Harvard Medical School Boston Massachusetts USA; ^3^ CPC Clinical Research University of Colorado School of Medicine Aurora Colorado USA

To the Editor,

We welcome and appreciate the comments made by Mei and colleagues about our recent publication, “Epidemiology of heart failure hospitalization in patients with stable atherothrombotic disease: Insights from the TRA 2°P‐TIMI 50 trial.” In this article, we show that the risk for hospitalization for heart failure (HHF) among patients with stable atherosclerosis is heterogeneous—with the highest risk in those with pre‐existing heart failure (adjusted OR 8.31, 95% CI 6.56–10.54), polyvascular disease (adjusted OR 2.68, 95% CI 1.94–3.70 for three vascular beds; adjusted OR 1.89, 95% CI 1.46–2.44 for two vascular beds), and type 2 diabetes (adjusted OR 2.55, 95% CI 2.01–3.24).

Mei and colleagues raise the concern that we may have overestimated the impact of pre‐existing heart failure on HHF in patients with stable atherosclerosis. The stated basis for their concern is that in a propensity matched analysis of 2,768 patients hospitalized for decompensated heart failure, Malik et al^1^ found that HHF within the previous 6 months portended a more modestly elevated risk of subsequent HHF over a 6‐month follow‐up period (HR 1.90, 95% CI 1.67–2.16 for HFpEF; HR 1.48, 95% CI 1.37–1.61 for HFrEF).

Although these analyses both support the prognostic relevance of prior heart failure when estimating risk of future adverse heart failure events, the difference in magnitude is not unexpected given the different populations. In the study by Malik et al,^1^ all patients had heart failure and the comparison was between those with and without prior hospitalization. In our study, we evaluated a large cohort with stable atherosclerosis, among whom only a small subset had a prior history of HF, and compared patients with and without any prior HF. In addition, the duration of follow up of 6 months in the former and approximately 3 years in the latter provides different observation time for events to occur.

Another difference between the studies is ascertainment and categorization of endpoints. In Malik and colleagues' propensity‐matched cohort study, HHF incidence was ascertained from Medicare claims. Billing codes have the potential for inflating event rates and diminishing specificity while adjudicated outcomes may reduce event rates and increase specificity. In fact, in our trial‐based analysis, despite a larger (~8‐fold) gradient of HHF risk, patients with pre‐existing heart failure actually had a lower cumulative HHF incidence in our study (3.8%/year) than was observed by Malik and colleagues among those with no HHF in the preceding 6 months (7.3%/year for HFpEF; 9.7%/year for HFrEF).

Mei and colleagues further propose that the value of pre‐existing heart failure in predicting incident HHF in our study may be confounded by the relatively high prevalence of atrial fibrillation among patients with heart failure. However, the number of patients with atrial fibrillation in the analysis was negligible, given that anticoagulation was an exclusion criterion for the TRA 2°P‐TIMI 50 trial.^2^

Lastly, Mei and colleagues point out that the reported null effect of vorapaxar on HHF incidence comes solely from a Cox proportional hazards model of time to first HHF, excluding the 116 recurrent HHF that occurred during 3‐year follow‐up. It is important to note that the allocation of vorapaxar in the analysis was randomized, and despite this, there was no numerical trend favoring vorapaxar over placebo in reducing the risk of first HHF (HR 1.15, 95% CI 0.93–1.42). Since first and recurrent heart failure events are related, a total events analysis would be unlikely to produce a change of the magnitude needed to show a benefit. With regard to the use of antithrombotic therapy in patients with heart failure, the 2021 European Society of Cardiology (ESC) Guidelines cite a subgroup analysis of the COMPASS trial which showed that low‐dose rivaroxaban has consistent benefit with respect to reducing MACE in patients with PAD and/or CAD and concomitant HFpEF or HFmrEF.^3^ The statement relates to the consistent relative risk reduction for MACE in this subgroup, which owing to its high baseline risk, has a large absolute benefit. Similarly, vorapaxar shows consistent benefit in patients with CAD and/or PAD with or without heart failure with respect to reducing MACE risk. Nonetheless, we did not observe a benefit in the exploratory endpoint of HHF. Similarly, in the COMMANDER trial, which recruited patients with heart failure and randomized them to rivaroxaban or placebo, the hazard ratio for HHF was 0.98 (95% CI 0.89–1.09).^4^ Taken together, these data support the lack of benefit of antithrombotic therapy in preventing heart failure events, at least for the drugs and durations studied.
